# Neuroimmune responses in neonatal hypoxic-ischemic encephalopathy: cellular roles, crosstalk, and therapeutic opportunities

**DOI:** 10.3389/fped.2026.1893178

**Published:** 2026-09-22

**Authors:** Shaoping He, Mengzao He, Shun Wu

**Affiliations:** Department of Neonatology, Hangzhou Women’s Hospital (Hangzhou Maternity and Child Health Care Hospital), Hangzhou, Zhejiang, China

**Keywords:** immune crosstalk, microglia, neonatal hypoxic-ischemic encephalopathy, neuroinflammation, precision immunotherapy

## Abstract

Neonatal hypoxic-ischemic encephalopathy (HIE) is traditionally viewed as a consequence of acute oxygen and blood flow deprivation followed by excitotoxicity, oxidative stress, and neuronal death. However, growing evidence indicates that HIE is also a dynamic neuroimmune disorder in which immune cells actively shape injury progression, tissue remodeling, and long-term neurodevelopmental outcome. Among these cells, microglia function as the central immune hub of the injured neonatal brain, rapidly responding to damage signals and interacting with neurons, myeloid cells, and adaptive immune components. Recent studies further show that microglial activation is not uniformly detrimental, but depends on timing, phenotype, and signaling context. In parallel, phagocytes and broader myeloid networks amplify neuroinflammation through transcriptional programs such as IRF5, while regulatory T cells contribute to outcome heterogeneity and sexual dimorphism. These findings highlight that immune crosstalk in neonatal HIE is dynamic, phase-dependent, and developmentally specific. Therapeutically, the field is moving beyond therapeutic hypothermia alone toward pathway-defined immunomodulation, extracellular vesicle- and mesenchymal stromal cell-based approaches, and combination strategies that reshape the immune microenvironment. This review summarizes the roles of immune cells in neonatal HIE, emphasizes the importance of cellular crosstalk and temporal immune-state transitions, and discusses emerging opportunities for precision neuroimmune targeting.

## Highlights

1. Neonatal HIE is a dynamic neuroimmune disorder rather than a purely metabolic-ischemic brain injury.
2. Microglia, myeloid cells, and Treg form a phase-dependent immune network that shapes injury, repair, and sex-specific outcomes.
3. Future treatment is shifting from hypothermia alone toward precision immunomodulation, including pathway-targeted therapy and MSC/EV-based combination strategies.

## 1 Introduction

Neonatal hypoxic-ischemic encephalopathy (HIE) has traditionally been viewed as a disorder initiated by acute oxygen and blood flow deprivation, leading to primary energy failure, excitotoxicity, oxidative stress, and rapid neuronal death (1–3). However, this framework is no longer sufficient to explain the full spectrum of pathological outcomes after neonatal hypoxia-ischemia, including neuronal loss, white matter injury, necrotic tissue loss, impaired maturation, and long-term neurodevelopmental deficits. Increasing evidence indicates that the initial metabolic collapse is followed by a prolonged secondary phase characterized by neuroinflammation, regulated cell death, impaired tissue repair, and altered developmental trajectories, all of which substantially shape neurological outcome (4, 5). In this context, these injury phenotypes are not simply passive consequences of the initial ischemic insult, but are shaped by evolving neuroimmune responses that differ across cell type, brain region, and developmental stage (6). This broader view is particularly important in neonates, because the immature brain responds to hypoxia-ischemia in a manner that differs from the adult brain (6, 7). The neonatal central nervous system is developmentally dynamic, with ongoing myelination, synaptic refinement, and immune maturation (8). As a result, post-ischemic injury unfolds within a tissue environment in which inflammatory signaling, phagocytic activity, and repair programs are tightly intertwined with normal developmental processes (9, 10). Therefore, inflammatory responses in neonatal HIE should not be understood merely as secondary epiphenomena; rather, they are integral determinants of whether the injured brain progresses toward further destruction or partial recovery. At present, therapeutic hypothermia (HT) remains the only established treatment for moderate to severe neonatal HIE (11). Yet its benefits are incomplete, and a substantial proportion of affected infants still experience death or long-term neurodevelopmental impairment. Importantly, the protective effect of HT is not limited to metabolic suppression. Experimental work shows that hypothermia also modulates the temporal and spatial polarization of myeloid cells after neonatal HI, suggesting that part of its benefit may derive from reshaping the post-ischemic immune milieu (11). This observation has major conceptual significance: it implies that immune regulation is already embedded within current standard care, and that future therapies may need to build on, rather than bypass, this immunological dimension. This therapeutic gap has driven growing interest in adjunctive strategies that target immune dysregulation more directly. Among the most promising approaches are mesenchymal stromal cell (MSC)-based therapies and extracellular vesicle (EV)-based interventions, both of which are thought to act in part through immune microenvironment modulation rather than through simple cell replacement (12, 13). The translational relevance of this direction is underscored by an early-phase clinical trial of allogeneic umbilical cord tissue-derived MSCs in neonates with HIE, which was designed to evaluate safety and preliminary efficacy on top of hypothermia (14). Together, these developments support the idea that neonatal HIE should be viewed not only as an acute metabolic insult but also as a targetable disorder of dysregulated neuroimmune repair.

A purely neuron-centered interpretation of HIE is increasingly inadequate. Although excitotoxicity, mitochondrial dysfunction, and oxidative stress remain central to early injury, they do not fully account for the striking heterogeneity in tissue loss, inflammatory burden, and long-term neurological outcome observed after similar hypoxic-ischemic insults (15). One major reason is that immune cells actively shape the post-injury environment. Rather than functioning as passive responders to cell death, they participate in cytokine release, phagocytosis, antigen presentation, trophic support, debris clearance, and the regulation of tissue remodeling (16–18). Consequently, whether injury expands or stabilizes depends not only on the severity of the original insult, but also on how resident and infiltrating immune cells behave over time. Microglia are central to this discussion because they are the earliest and most abundant immune sentinels in the neonatal brain (19). However, recent studies show that their role cannot be reduced to a simple pro-inflammatory stereotype. In a microglia-depleted mouse model, depletion aggravated infarct volume and neuronal apoptosis after neonatal HI, particularly in males, indicating that microglia can also exert protective effects under certain developmental and temporal conditions (20). These findings argue strongly against blanket microglial suppression and instead favor a more nuanced framework in which timing, phenotype, and local context determine whether microglia amplify injury or facilitate adaptation. An immune-cell-centered view is also needed because HIE-associated inflammation extends beyond resident microglia. Phagocytes and other myeloid cells contribute to inflammatory amplification and repair programming, and their behavior is regulated by defined molecular switches (13). For example, IRF5 signaling in phagocytes has been shown to be detrimental in neonatal HIE, with phagocytic IRF5 deletion reducing pro-inflammatory responses and attenuating injury (12). This indicates that the inflammatory landscape of HIE is best understood as a coordinated myeloid network rather than as an isolated microglial reaction (21). Furthermore, emerging evidence suggests that adaptive immune mechanisms also contribute to neonatal HIE (22). Regulatory T cells (Treg) have been implicated in the sexual dimorphism of neonatal hypoxic-ischemic brain injury, highlighting that immune regulation in this setting is not restricted to innate immunity alone (23). This is particularly important because it links immune-cell composition and function to one of the most reproducible biological features of neonatal HI: sex-dependent differences in vulnerability and recovery (24). Therefore, an immune-cell-centered framework not only broadens mechanistic understanding but also helps explain clinically relevant heterogeneity that cannot be captured by neuron-centric models. Finally, the neonatal immune response cannot simply be extrapolated from adult ischemic stroke. The immature brain develops within a distinct neuroimmune environment, with different basal microglial states, distinct peripheral immune interactions, and ongoing developmental programs that alter the consequences of inflammation (25). Thus, immune responses in neonatal HIE are developmentally specific, and any meaningful synthesis of the field must account for these age-dependent biological features.

In light of these considerations, this review adopts an immune-cell-centered perspective to examine the pathophysiology and therapeutic opportunities of neonatal HIE (22). We first focus on microglia and myeloid cells as the principal innate immune drivers of the post-ischemic response, emphasizing their dual roles in injury propagation and tissue protection (25). Rather than presenting these cells as uniformly harmful, we discuss how their effects depend on activation state, signaling context, and temporal phase after injury (26). We then summarize emerging evidence for adaptive immune involvement, with particular attention to regulatory T cells and sex-dependent immune differences. This aspect is increasingly important because it expands the conceptual framework of neonatal HIE beyond innate neuroinflammation and toward a more integrated model of developmental neuroimmunology (22). A further objective of this review is to highlight cellular crosstalk within the injured neonatal brain. Immune cells do not operate in isolation; instead, they interact dynamically with neurons, astrocytes, oligodendrocyte lineage cells, endothelial cells, and the blood-brain barrier. These interactions shape not only acute injury severity but also subsequent repair, white matter maturation, and long-term neurodevelopmental outcome. Accordingly, understanding HIE requires attention to immune networks rather than to single-cell effects alone. Finally, we review therapeutic strategies aimed at immune modulation in neonatal HIE, including the immunoregulatory effects of hypothermia, pathway-based inhibition of injurious inflammatory signaling, and the emerging use of MSC- and EV-based interventions (27, 28). We also consider the translational trajectory of these approaches, from mechanistic studies in experimental models to early-phase clinical testing. Through this structure, we aim to provide an updated synthesis of how immune cells drive, shape, and potentially resolve neonatal hypoxic-ischemic brain injury, and to outline the key challenges that must be addressed to move toward precision immunotherapy in this field.

In this review, the term “brain injury” is used to encompass several distinct but overlapping pathological phenotypes of neonatal HIE, including acute neuronal loss, diffuse white matter injury, cystic or necrotic tissue loss, and impaired maturation of oligodendrocyte, synaptic, and neural circuit development. These phenotypes differ in timing, anatomical distribution, cellular vulnerability, and immune involvement. For example, acute neuronal loss is closely linked to excitotoxicity, mitochondrial dysfunction, regulated cell death, and early inflammatory amplification, whereas diffuse white matter injury involves oligodendrocyte lineage vulnerability, microglia-mediated inflammation, oxidative stress, and impaired myelination. Cystic necrosis reflects severe focal tissue destruction and subsequent inflammatory clearance, while impaired maturation reflects longer-term disruption of developmental programs after the initial insult. Therefore, immune pathways discussed below are interpreted in relation to specific injury phenotypes rather than as generic mechanisms of “brain injury.”

## 2 Microglia as spatially and developmentally patterned immune hubs in neonatal HIE

To avoid treating neonatal HIE as a single pathological entity, this section links microglial and immune-mediated pathways to specific injury phenotypes, including acute neuronal loss, white matter injury, necrotic tissue loss, and impaired maturation. Immune responses in neonatal HIE involve multiple resident and infiltrating cell populations rather than a single inflammatory cell type (29, 30). Microglia act as the earliest immune sentinels and central coordinators, whereas phagocytes and broader myeloid cells amplify inflammatory expansion through defined transcriptional programs (25, 26). In addition, regulatory T cells and innate–adaptive immune interactions contribute to immune balance, outcome heterogeneity, and sex-dependent injury responses. These major immune cell populations and their functional implications are summarized in Table 1.

**Table 1:** Immune cell populations and their roles in neonatal HIE.

Immune cell type	Main role in neonatal HIE	Representative mechanisms/functions	Key message for the review	Representative references
Microglia	Earliest and central immune responders	Rapid sensing of DAMPs, cytokine release, phagocytosis, inflammasome activation, pyroptosis	Microglia are the central immune hub of neonatal HIE and can be either protective or detrimental depending on timing and phenotype	(16, 20, 54)
Phagocytes/myeloid cells	Amplifiers of neuroinflammation	IRF5-dependent pro-inflammatory programming, coordination of inflammatory expansion	Injury progression is shaped by a broader myeloid immune network rather than microglia alone	(12)
Microglia/macrophage polarized states	Dynamic regulators of inflammatory balance	Temporal and spatial polarization changes after HI; modulation by hypothermia	Standard therapy already exerts immunomodulatory effects by reshaping myeloid polarization	(13)
Regulatory T cells (Treg)	Adaptive immune regulators of injury heterogeneity	Modulation of inflammatory tone, immune restraint, contribution to sex-specific outcome differences	Adaptive immunity is relevant in neonatal HIE and should not be ignored	(23)
Innate–adaptive immune interface	Crosstalk between microglia and adaptive immune cells	Cytokine-mediated regulation of local immune phenotype	Immune responses in neonatal HIE are networked rather than isolated by cell type	(20, 23)

### 2.1 Developmental and spatial context of microglial responses

Before discussing microglial activation after neonatal hypoxic-ischemic injury, it is important to recognize that microglia in the neonatal brain do not exist as a uniform or developmentally static population. During early brain development, microglia participate in neurogenesis, vascular development, synaptic remodeling, myelination-related processes, and the establishment of local neural circuits (16, 31, 32). Their density, morphology, transcriptional state, and functional interactions vary across brain regions and developmental stages. Therefore, hypoxic-ischemic injury is superimposed on a spatially organized and developmentally active microglial system rather than on a silent or immunologically neutral baseline.

Microglia are widely regarded as the first immune cells to sense and react to hypoxic-ischemic injury in the neonatal brain (33). As the resident innate immune sentinels of the central nervous system, they rapidly detect danger-associated signals released from injured neurons, dying oligodendrocyte lineage cells, disrupted synapses, and damaged vascular elements (34). Once activated, microglia initiate an inflammatory cascade through cytokine release, phagocytic responses, oxidative signaling, and interaction with other brain-resident and infiltrating immune cells (31, 35, 36). In this sense, microglia occupy a central position at the interface between primary metabolic injury and secondary inflammatory propagation. This early and central role, however, should not be mistaken for a purely detrimental function. A major advance in the field has been the recognition that microglial responses in neonatal HIE are strongly dependent on timing, activation state, and developmental context (37, 38). In microglia-depleted neonatal mice, hypoxic-ischemic injury was aggravated rather than alleviated, with increased neuronal apoptosis and more severe tissue injury, particularly in males (20). These findings indicate that at least a subset of microglial responses may restrain injury expansion, support debris clearance, or preserve tissue homeostasis during the early post-insult period. Thus, the key question is not whether microglia are “good” or “bad,” but when and under what conditions they shift from adaptive surveillance to maladaptive inflammatory amplification. At the same time, microglia are clearly capable of driving tissue damage once inflammatory programs become excessive or sustained. One important mechanism is pyroptosis, a lytic and highly inflammatory form of programmed cell death that links innate immune sensing to the release of potent cytokines (39–41). Evidence from neonatal HIE models indicates that microglia can undergo pyroptotic activation and thereby amplify local inflammatory injury. This duality is conceptually important for the whole review: microglia are not merely passive inflammatory markers, nor are they uniformly destructive. Rather, they are dynamic regulators whose function can vary from transient protection to active execution of secondary brain injury.

Taken together, current evidence supports viewing microglia as regionally and developmentally patterned immune hubs in neonatal HIE. They are among the earliest responders and important coordinators of downstream neuroinflammation, but their functions are shaped by local brain-region vulnerability, developmental stage, surrounding neural and vascular elements, and the timing of injury. This perspective avoids treating microglia as a homogeneous population and helps explain why the same injury may produce different inflammatory and repair responses across gray matter, white matter, hippocampal, cortical, or periventricular regions.

### 2.2 Hypoxic-ischemic injury triggers microglial pyroptosis and amplifies secondary inflammation

Pyroptosis should be interpreted as a downstream inflammatory cell-death mechanism triggered by neonatal hypoxic-ischemic injury rather than as an initiating cause of HIE (18, 39). After hypoxia-ischemia, primary energy failure, mitochondrial dysfunction, oxidative stress, calcium imbalance, and membrane disruption promote the release of danger-associated molecular patterns and inflammatory mediators from injured neural and vascular cells. These injury-derived signals can activate pattern-recognition and inflammatory pathways in microglia, including TLR/NF-κB-related priming of inflammasome components and subsequent NLRP3/caspase-1/GSDMD-dependent pyroptotic signaling (39, 42–45). As a result, microglial pyroptosis promotes the release of IL-1β, IL-18, and other inflammatory mediators, thereby amplifying secondary neuroinflammation and neuronal injury.

Lv and colleagues provided foundational evidence that microglia-mediated pyroptosis plays a crucial role in neonatal HIE. Their work linked hypoxic-ischemic injury to increased expression of pyroptosis-associated mediators in microglia, supporting the idea that inflammatory cell death is not a downstream bystander event but a mechanistically important contributor to brain injury progression (46). These observations indicate that microglial pyroptosis is not merely a downstream biochemical signature, but a mechanistically relevant contributor to secondary brain injury. Accordingly, microglial pyroptosis may represent a mechanism through which innate immune activation contributes to secondary structural brain injury.

Chen and colleagues added an important mechanistic layer by connecting microglial inflammation in neonatal HIE to the NLRP3 inflammasome axis. They showed that miR-374a-5p suppresses microglial inflammatory signaling by targeting Smad6 and inhibiting NLRP3-related activation, thereby reducing the release of pro-inflammatory factors (47). These results place inflammasome regulation upstream of the inflammatory microglial phenotype and suggest that NLRP3-dependent signaling is one of the molecular gateways through which HI promotes microglial pathogenicity. It also reinforces the idea that post-transcriptional regulators can shape whether microglia enter an injury-amplifying state. From a translational perspective, Tao and colleagues further strengthened this framework by showing that Echinatin reduced cerebral edema, infarct volume, long-term behavioral impairment, microglial activation, inflammatory factor expression, and pyroptosis markers in neonatal hypoxic-ischemic brain damage (48). Mechanistically, these protective effects were associated with inhibition of the TLR4/NF-κB pathway. Together with the above evidence, these data link upstream TLR4/NF-κB signaling to downstream pyroptotic injury and indicate that pharmacologic interruption of this axis can attenuate both inflammation and tissue damage. It therefore supports the idea that microglial pyroptosis is not only mechanistically relevant but also therapeutically targetable.

Taken together, these studies support a coherent pathogenic chain in neonatal HIE: hypoxic-ischemic injury activates innate danger signaling, promotes inflammasome engagement and pyroptotic machinery in microglia, drives release of cytokines such as IL-1β and IL-18, and thereby amplifies secondary tissue injury. This framework also helps explain why interventions aimed at TLR4/NF-κB signaling, inflammasome activation, or pyroptotic execution may produce neuroprotection even when the initial ischemic insult has already occurred (48–52). In this sense, microglial pyroptosis is not simply one inflammatory pathway among many, but one of the clearest mechanistic bridges between immune activation and ongoing brain destruction in neonatal HIE.

### 2.3 Upstream signaling programs that drive microglial inflammation

The pathogenic behavior of microglia in neonatal HIE is not random, but is organized by a set of upstream inflammatory signaling programs that translate hypoxic-ischemic stress into sustained immune activation. Among the best-characterized pathways are NF-κB, p38-MAPK, and TLR4-related signaling, which function as central hubs connecting danger sensing to cytokine production, oxidative stress, and inflammatory gene expression (43, 45, 53). In the immature brain, these pathways appear to be especially important because they influence not only the magnitude of acute inflammation but also the broader balance between destructive and reparative microglial states.

Selective inhibition of NF-κB in microglia attenuated hypoxic-ischemic injury in the immature brain, reduced microglial activation and pro-inflammatory cytokine expression, and improved tissue outcome. These results support the idea that NF-κB is not merely a marker of microglial activation, but a functional driver of microglia-mediated injury (54). This finding is highly relevant to neonatal HIE because it suggests that the inflammatory contribution of microglia can be uncoupled from their homeostatic roles by targeting pathway-defined pathogenic programs rather than eliminating the cells themselves. Aoki and colleagues further expanded this framework by identifying lectin-like oxidized low-density lipoprotein receptor-1 (LOX-1) as an upstream mediator of inflammatory activation in microglial cells under hypoxic-ischemic conditions. Their work linked LOX-1 to p38-MAPK and NF-κB activation, positioning it as a danger-sensing node that helps convert ischemic stress into a pro-inflammatory microglial phenotype (53). Conceptually, this is important because it shifts the discussion from downstream cytokines to the upstream receptors and signal-transduction events that initiate inflammatory amplification. These observations also integrate well with pharmacologic evidence. The protective effect of Echinatin in neonatal hypoxic-ischemic brain damage has been linked to inhibition of the TLR4/NF-κB pathway together with reduced inflammatory injury and pyroptosis. Taken together, the available evidence supports a model in which multiple upstream sensors and signaling modules converge on common inflammatory execution pathways in microglia. This convergence is therapeutically meaningful: it implies that microglial pathogenicity in neonatal HIE may be more effectively controlled by interrupting defined signaling circuits than by broad, nonspecific suppression of inflammation.

### 2.4 MicroRNAs are altered by HIE and regulate microglial inflammatory responses

MicroRNAs in neonatal HIE should be interpreted as both injury-responsive molecules and functional regulators of secondary neuroinflammation. On the one hand, hypoxic-ischemic injury can alter microRNA expression through hypoxia-responsive signaling, oxidative stress, mitochondrial dysfunction, inflammatory activation, and cellular stress responses (47, 55–58). On the other hand, these altered microRNAs may feed back to influence disease progression by regulating microglial activation, inflammasome signaling, cytokine production, and injury-associated inflammatory amplification. Therefore, microRNAs are not presented here as initiating causes of HIE, but as post-transcriptional regulators that link the hypoxic-ischemic environment to later immune-state remodeling.

Li and colleagues identified miR-210 as a regulator of microglial activation in neonatal HIE. Their study showed that miR-210 promotes microglia-mediated neuroinflammation, supporting the broader idea that hypoxic-ischemic injury reprograms immune responses not only through transcriptional activation but also through microRNA-mediated control (56). This is especially relevant in neonatal HIE because miR-210 is closely linked to hypoxic signaling, making it a plausible molecular bridge between the primary ischemic environment and the later inflammatory response.

By contrast, Chen and colleagues demonstrated that miR-374a-5p has an anti-inflammatory role by suppressing microglial activation through a Smad6/NLRP3-related mechanism (47). This finding adds an important nuance to the field: microRNAs in neonatal HIE are not uniformly pro-inflammatory, but instead form a regulatory network with both injury-promoting and injury-limiting components. Such studies support the view that microglial pathogenicity is actively tuned by competing post-transcriptional programs rather than passively dictated by the severity of the insult alone. A further example comes from the study of miR-384, which was reported to be deregulated in neonatal HIE and to alleviate microglia-mediated neuroinflammation while also showing biomarker potential. This dual role is conceptually valuable for a review because it links mechanistic biology to clinical translation. In other words, some non-coding RNAs may not only participate in shaping microglial inflammatory states but may also help stratify disease severity or identify therapeutic windows. That possibility is particularly attractive in neonatal HIE, where treatment timing is critical and reliable immune-state biomarkers remain limited.

Overall, current evidence supports a bidirectional model in which neonatal hypoxic-ischemic injury alters microRNA expression, and these altered microRNAs subsequently regulate microglial immune responses through post-transcriptional control of inflammatory pathways. In this model, microRNAs function as injury-responsive regulators rather than primary causes of HIE. Their main relevance lies in linking the hypoxic-ischemic environment to downstream immune remodeling, inflammasome activity, cytokine release, and potential biomarker development.

### 2.5 Microglia are not purely detrimental: timing, phenotype, and activation threshold matter

Importantly, this threshold is unlikely to be identical across all brain regions, because microglial activation is shaped by local developmental programs, regional circuit formation, white matter vulnerability, and differences in neuronal or oligodendrocyte maturation. One of the most important conceptual shifts in the field is the recognition that microglia in neonatal HIE cannot be classified as uniformly harmful. The same cell population may exert divergent effects depending on the time after injury, the local tissue environment, and the specific signaling context in which activation occurs (59). This is particularly relevant in the developing brain, where microglia are already involved in physiological synaptic remodeling, debris clearance, and support of neural circuit maturation even before injury occurs (60). As a result, hypoxic-ischemic activation is superimposed on pre-existing developmental functions rather than occurring in a biologically neutral background. This dual role can be conceptualized as a threshold-dependent response. At low to moderate levels, or during early and well-controlled activation, microglia may contribute to debris clearance, injury containment, synaptic remodeling, and immune resolution (43, 61–64). However, when activation exceeds a certain intensity or persists beyond the adaptive phase, microglia may shift toward an injury-amplifying state characterized by excessive cytokine release, oxidative stress, inflammasome activation, pyroptosis, and neuronal damage. Thus, the biological effect of microglia in neonatal HIE depends not only on whether they are activated, but also on the magnitude, duration, and timing of that activation.

The microglia-depletion study by Tsuji and colleagues remains especially important in this regard because it demonstrated that removing microglia before neonatal HI aggravated rather than relieved brain injury (20). These findings argue strongly against simplistic therapeutic strategies based on global microglial ablation. Instead, they support a more precise model in which certain microglial activities may be beneficial early on, for example by limiting damage spread, clearing injured cellular material, or containing inflammatory disorganization, whereas prolonged or dysregulated activation later becomes pathogenic. This framework also helps unify apparently conflicting findings in the literature. Studies showing that microglia contribute to pyroptosis, inflammasome activation, and NF-κB-dependent inflammation do not contradict evidence for protective microglial functions; rather, together they suggest that microglia move across functional states over time (45, 46, 65, 66). The biological challenge, therefore, is not to decide whether microglia should be inhibited in general, but to determine which microglial programs should be preserved, which should be redirected, and which should be blocked at specific phases of neonatal HIE. For this reason, microglia are best viewed not as a single pathogenic entity but as a dynamic immune hub whose phenotype evolves during injury progression and repair (13, 66, 67). This has major therapeutic implications. The most promising strategies are likely to be those that modulate microglial state transitions, restrain defined pro-inflammatory pathways, and preserve reparative functions, rather than those that indiscriminately suppress the entire microglial compartment. Such a state-specific view is especially important in neonatal HIE, where immune responses are tightly interwoven with developmental processes and where mistimed intervention could theoretically disrupt both repair and maturation. Overall, microglial responses in neonatal HIE are shaped by an integrated regulatory network involving inflammasome activation, NF-κB-related inflammatory signaling, upstream danger-sensing pathways, transcriptional programming, and ncRNA-mediated control. These mechanisms explain why microglia may shift between injury-amplifying and tissue-protective states depending on timing and phenotype. The major signaling pathways and regulatory axes are summarized in Table 2, and the microglia-centered immune hub model is illustrated in Figure 1.

**Table 2:** Key signaling pathways regulating immune activation and neuroinflammation in neonatal HIE.

Pathway/regulator	Main immune cell target	Functional consequence	Translational implication	Representative references
NLRP3/caspase-1/GSDMD pyroptosis axis	Microglia	Promotes inflammatory cell death and release of IL-1β/IL-18, amplifying secondary injury	Supports targeting pyroptosis/inflammasome pathways	(16, 47)
NF-κB signaling	Microglia	Drives pro-inflammatory activation and cytokine production	Microglial NF-κB is a pathway-defined therapeutic target	(54)
TLR4/MyD88/NF-κB axis	Microglia/innate immune cells	Links danger sensing to inflammatory amplification and pyroptosis	Pharmacologic inhibition may attenuate both inflammation and tissue damage	(48)
LOX-1/p38-MAPK/NF-κB axis	Microglia	Upstream inflammatory activation under hypoxic-ischemic conditions	Identifies receptor-level danger sensing as a targetable node	(53)
IRF5-dependent transcriptional programming	Phagocytes/myeloid cells	Biases myeloid cells toward detrimental pro-inflammatory phenotypes	Suggests myeloid reprogramming as a therapeutic strategy	(12)
miR-210-related regulation	Microglia	Promotes microglia-mediated neuroinflammation	Indicates that non-coding RNAs may shape immune-state transitions	(56)
miR-374a-5p/Smad6/NLRP3 regulation	Microglia	Restrains inflammasome-related inflammatory activation	Supports ncRNA-based fine-tuning of microglial phenotypes	(47)
miR-384-related regulation	Microglia	Suppresses neuroinflammation and may serve as a biomarker	Links mechanism with biomarker development	(64)

**Figure 1:**
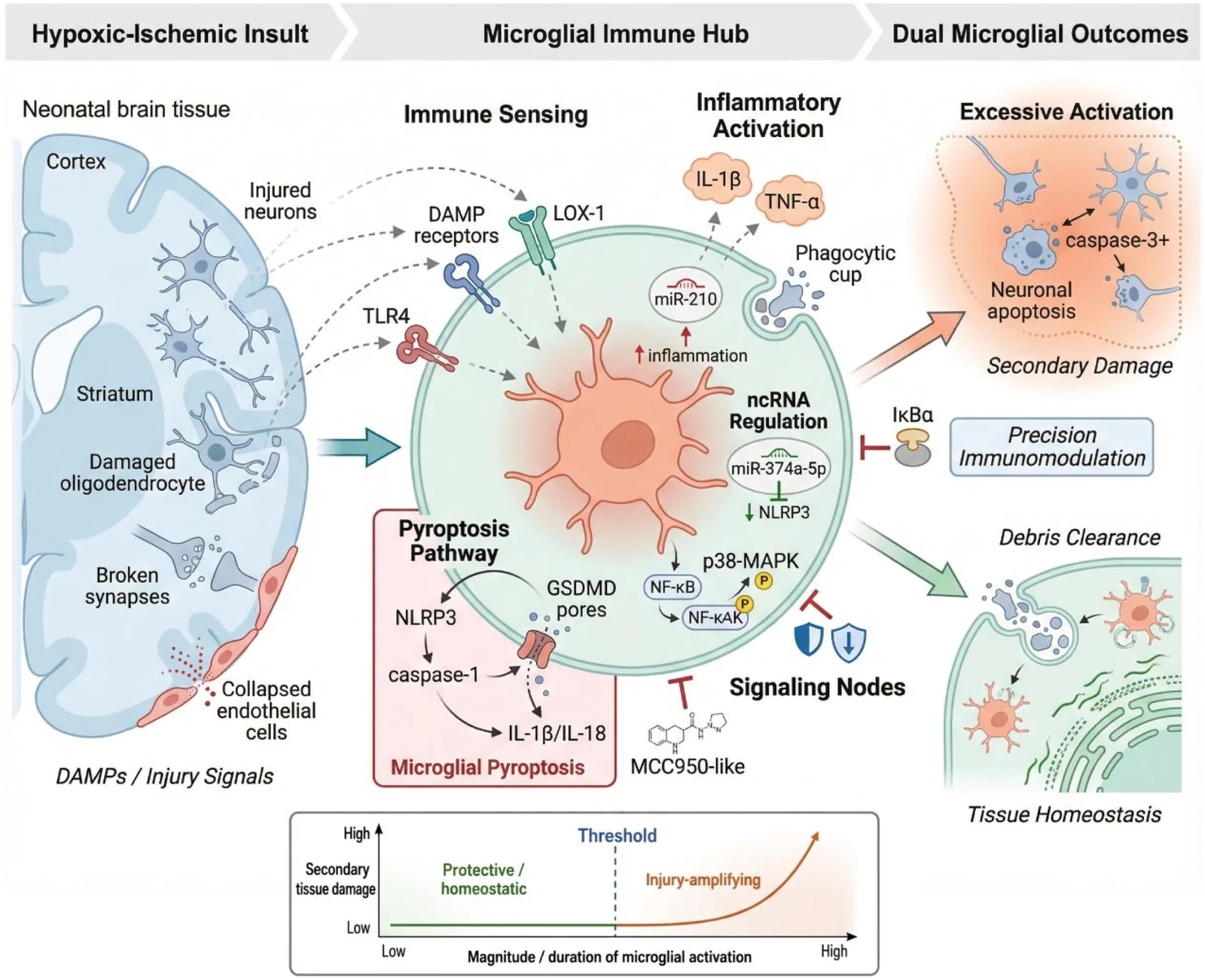
Microglia as the central immune hub in neonatal hypoxic-ischemic encephalopathy. Hypoxic-ischemic injury triggers the release of danger signals from damaged neural and vascular elements, leading to rapid microglial activation. Activated microglia integrate inflammatory pathways, pyroptosis-related signaling, and ncRNA regulation, thereby shaping both detrimental and protective responses. Depending on timing and phenotype, microglia may amplify neuroinflammation and secondary brain injury or contribute to debris clearance, tissue homeostasis, and injury containment.

## 3 Myeloid cells and phagocytes as amplifiers of neuroinflammation

### 3.1 Beyond microglia: phagocytes orchestrate inflammatory expansion

Although microglia are the most intensively studied immune cells in neonatal HIE, the post-ischemic inflammatory response cannot be fully explained by resident microglia alone (13, 30, 68). Hypoxic-ischemic injury unfolds within a broader myeloid immune network in which microglia, macrophage-like phagocytes, and other myeloid populations collectively shape the balance between inflammatory injury and tissue adaptation (13, 47). This broader view is important because it shifts the focus from a single-cell perspective to a coordinated immune system response, in which multiple phagocytic populations contribute to cytokine production, debris clearance, antigen processing, and repair-associated remodeling (68, 69).

In this network, phagocytes are not passive scavengers. Rather, they help determine whether the injured neonatal brain enters a state of escalating neuroinflammation or transitions toward partial containment and recovery (66). Their contribution is especially relevant during the secondary phase of injury, when inflammatory mediators, dying cells, and tissue-derived danger signals continue to accumulate (70, 71). Under these conditions, myeloid cells act as both sensors and effectors, receiving injury-associated cues and converting them into broader inflammatory programs that influence surrounding neurons, oligodendrocyte lineage cells, and vascular elements (71). This makes the phagocyte compartment a key amplifier of injury progression rather than a mere downstream consequence of tissue damage. A central insight from recent work is that these phagocytic responses are governed by defined transcriptional programs rather than by nonspecific activation alone. In particular, interferon regulatory factor 5 (IRF5) has emerged as a major determinant of pro-inflammatory myeloid behavior in neonatal HIE (12). These data suggest that inflammatory amplification in the injured neonatal brain is not simply the result of cell accumulation, but is shaped by lineage-specific immune programming. Accordingly, the myeloid compartment can be viewed not only as a source of inflammation, but also as a therapeutically tunable system whose transcriptional state may influence disease severity. Taken together, these observations support an expanded model of neonatal HIE in which post-ischemic inflammation is orchestrated by a myeloid immune network rather than by microglia in isolation. This broader framework provides a necessary bridge between resident neuroimmune responses and later discussions of therapeutic immune modulation.

Multiple signaling and transcriptional pathways may regulate myeloid activation after neonatal hypoxic-ischemic injury. IRF5 is discussed below not as an exclusive or universal pathway, but as a representative example of transcription factor-dependent myeloid programming (12). This choice is based on direct evidence linking phagocytic IRF5 signaling to pro-inflammatory responses and injury severity in neonatal HIE. By focusing on IRF5, this section illustrates how the phenotype of phagocytes and broader myeloid cells may be shaped by defined transcriptional programs rather than by nonspecific activation alone.

### 3.2 IRF5-dependent myeloid programming as a representative transcriptional mechanism

Among the molecular programs that regulate myeloid activation, IRF5 provides a representative example of transcription factor-dependent inflammatory programming in neonatal HIE (13, 72). Al Mamun and colleagues showed that phagocytic IRF5 signaling contributes to detrimental inflammatory responses after neonatal hypoxic-ischemic injury. Deletion of phagocytic IRF5 reduced pro-inflammatory responses, enhanced anti-inflammatory signaling, and attenuated brain injury (12). These findings indicate that myeloid-mediated inflammation is not determined only by cell accumulation, but also by the transcriptional state of phagocytic cells.

This is highly relevant for a review centered on immune cells because it suggests that myeloid cells in neonatal HIE are not merely harmful due to their presence, but due to how they are programmed. Such a distinction has important therapeutic consequences. If detrimental inflammation is encoded through a specific transcriptional network, then intervention may be possible without indiscriminately suppressing the entire myeloid compartment. This is biologically preferable in the developing brain, where complete immune silencing could interfere with physiological clearance and repair processes (73). More broadly, the IRF5 story supports the idea that “myeloid reprogramming” deserves to be treated as an independent mechanistic layer in neonatal HIE. It provides a framework for understanding how inflammatory expansion is sustained and why some phagocytic responses become maladaptive (12). It also opens the possibility that future therapies may work not by blocking all inflammation, but by redirecting myeloid cells away from destructive transcriptional states and toward more reparative ones.

### 3.3 Therapeutic hypothermia remains standard care and may modulate myeloid polarization

Therapeutic hypothermia is currently the only established treatment for moderate to severe neonatal HIE and remains the foundation of clinical management (74–76). Its clinical use should not be minimized, because no immune-targeted intervention has yet replaced hypothermia in routine neonatal care. Traditionally, the protective effects of hypothermia have been explained mainly by reduced metabolic demand, attenuation of excitotoxicity, decreased oxidative stress, and modulation of cell death-related pathways. Experimental evidence further suggests that hypothermia may also influence the temporal and spatial dynamics of microglia/macrophage polarization after neonatal hypoxic-ischemic injury. These immunological observations should be interpreted cautiously and clinically. They do not negate the established role of hypothermia, nor do they imply that immune modulation is the dominant mechanism of its benefit. Rather, they suggest that the neuroprotective effects of hypothermia may include partial modulation of the post-ischemic inflammatory milieu. From a translational perspective, this supports the development of adjunctive therapies that complement standard hypothermia by targeting persistent inflammation or impaired repair, instead of replacing hypothermia as the current treatment backbone.

Seitz and colleagues showed that hypothermia influences microglia/macrophage polarization patterns after neonatal hypoxic-ischemic injury (13). These findings suggest that HT may influence the inflammatory architecture of the injured brain in addition to its established effects on metabolic suppression, excitotoxicity, oxidative stress, and cell death. In practical terms, standard therapy may already be altering how myeloid cells distribute, activate, and interact with damaged tissue. This supports a more integrated interpretation of neonatal HIE treatment, in which neuroprotection and immunomodulation are not distinct categories but overlapping processes (77). The implications are substantial. First, they reinforce the concept that myeloid cells are central to disease evolution, since a therapy known to improve outcome also alters their behavior. Second, they suggest that adjunctive therapies should be designed with hypothermia in mind, not simply added on top of it in a biologically disconnected way. If HT already biases the injured brain toward a particular myeloid state, then future immune-targeted interventions may be most effective when they complement or extend that shift rather than counteract it. This point also provides a natural transition to the next section. If innate immune cells such as microglia and phagocytes can be reshaped by standard treatment, then it becomes important to ask whether adaptive immune components are also involved in determining disease trajectory (13). Together, these findings indicate that post-ischemic inflammation in neonatal HIE is organized by a broader myeloid immune network rather than by microglia alone. IRF5-dependent phagocyte programming promotes inflammatory amplification, whereas therapeutic hypothermia can partially reshape myeloid polarization. This network-based model of myeloid immune regulation is shown in Figure 2.

**Figure 2:**
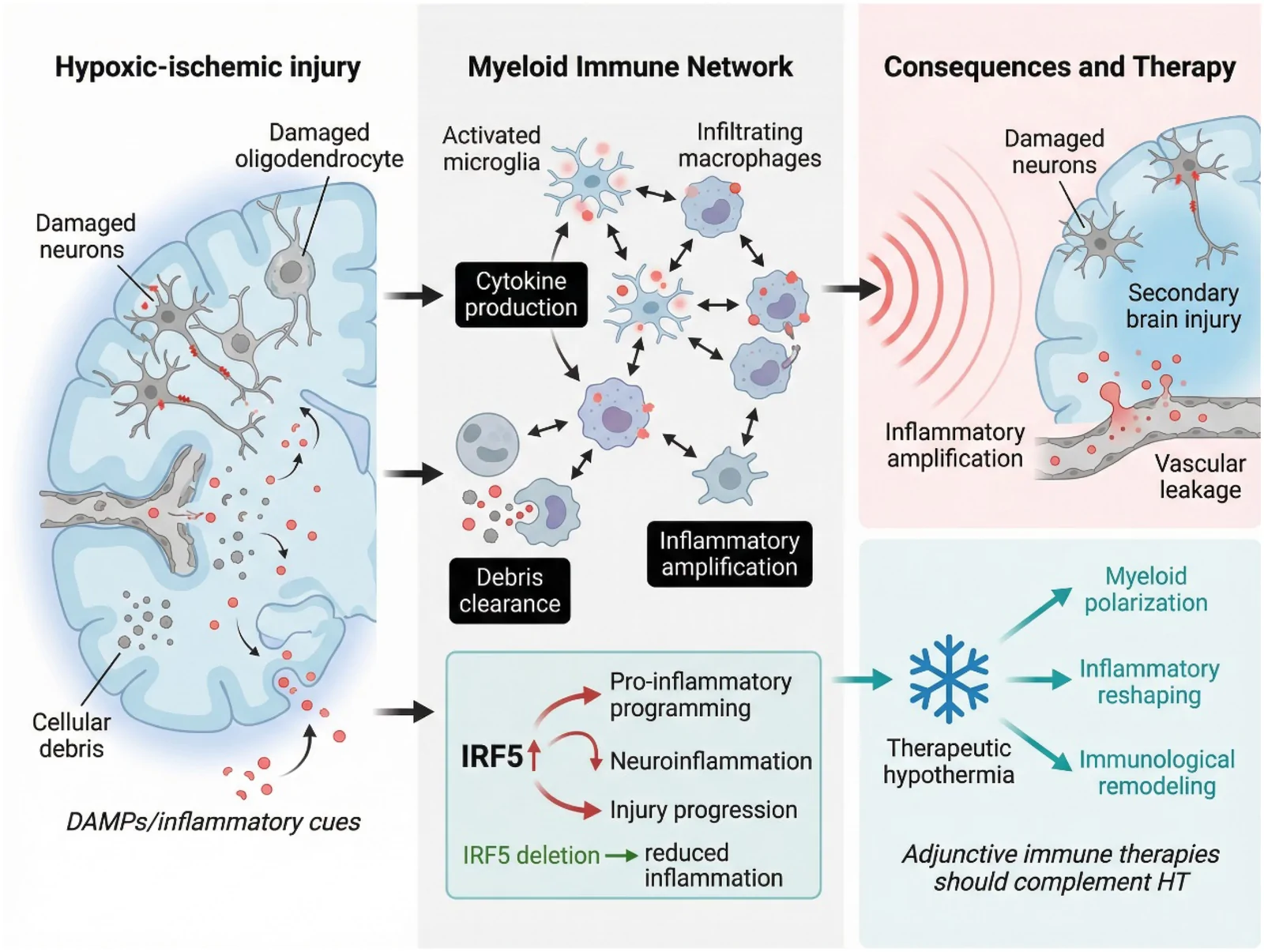
Myeloid cells and phagocytes as amplifiers of neuroinflammation in neonatal hypoxic-ischemic encephalopathy. Hypoxic-ischemic injury activates a broader myeloid immune network composed of resident microglia and phagocytic myeloid cells. Through IRF5-dependent pro-inflammatory programming, these cells amplify neuroinflammation and secondary tissue injury. Therapeutic hypothermia partially reshapes myeloid polarization and inflammatory organization, highlighting that post-ischemic immune responses are network-based and therapeutically modifiable.

### 3.4 Innate immunity as the first-line neuroimmune response

Taken together, the microglial and myeloid responses discussed above represent the major components of innate immunity in neonatal HIE. After hypoxic-ischemic injury, resident microglia rapidly sense tissue-derived danger signals and initiate inflammatory signaling, phagocytosis, oxidative stress responses, and inflammasome-related activation. In parallel, peripheral monocytes, macrophage-like phagocytes, neutrophils, and broader myeloid populations may contribute to inflammatory amplification, debris clearance, and tissue remodeling. Therefore, innate immunity in neonatal HIE should be understood as a coordinated resident–peripheral immune network rather than as an isolated microglial reaction. Importantly, innate immune activation is not uniformly detrimental. Microglia and myeloid cells may amplify secondary injury through NLRP3 inflammasome activation, TLR/NF-κB signaling, LOX-1/p38-MAPK/NF-κB activation, pyroptosis, and IRF5-dependent inflammatory programming (51, 78–81). However, these cells may also contribute to injury containment, debris clearance, immune resolution, and repair-oriented remodeling. This dual role explains why broad suppression of innate immunity may be inappropriate in the developing brain. Instead, future therapeutic strategies should aim to selectively restrain injurious innate immune programs while preserving protective and reparative functions.

## 4 Adaptive immunity and sex-dependent immune responses

### 4.1 Adaptive immune components are increasingly recognized in neonatal HIE

For many years, the immunopathology of neonatal HIE was interpreted mainly through the lens of innate immunity, with microglia and myeloid inflammation occupying the center of attention (22, 82). This emphasis was understandable, given the rapid activation of resident immune cells and the strong inflammatory response that follows hypoxic-ischemic injury (83). Yet recent studies indicate that this framework is incomplete. Adaptive immune components, particularly T cells and regulatory T cells (Treg), are increasingly recognized as contributors to disease evolution in neonatal HIE (84). This shift is important for several reasons. First, it broadens the mechanistic landscape of neonatal HIE beyond innate neuroinflammation and suggests that longer-range immune regulation may influence tissue outcome. Second, it introduces the possibility that immune-cell composition and function help explain heterogeneity in injury severity and recovery. Third, it aligns neonatal HIE with a wider neuroimmunology framework in which crosstalk between innate and adaptive compartments shapes the trajectory of brain injury (84). Rather than treating adaptive immunity as irrelevant because of neonatal immune immaturity, the emerging literature suggests that developmental immune specificity itself may be a central part of the disease (26).

Beckmann and colleagues provided particularly important evidence by showing that regulatory T cells contribute to sexual dimorphism in neonatal hypoxic-ischemic brain injury (23). These data implicate Treg in neonatal HI and connect adaptive immune regulation with sex-dependent differences in vulnerability and therapeutic response. In doing so, it moves adaptive immunity from the periphery to a meaningful position within the pathophysiology of neonatal HIE.

### 4.2 Regulatory T cells contribute to outcome heterogeneity

Regulatory T cells are best known for their capacity to restrain excessive immune activation and preserve immune homeostasis (85). In neonatal HIE, however, their significance likely extends beyond a simple anti-inflammatory label (23, 84). Treg may influence the intensity, duration, and spatial organization of post-ischemic inflammation, while also shaping the broader repair environment in the injured brain (86). This is especially plausible in a developmental context, where inflammatory control is closely tied to ongoing maturation, tissue remodeling, and long-term functional outcome (87).

The study by Beckmann and colleagues supports the idea that Treg contribute meaningfully to outcome heterogeneity after neonatal HI. Their involvement suggests that differences in adaptive immune regulation may help determine why seemingly similar injuries can lead to different pathological and developmental trajectories (23). This is a major conceptual advance because it implies that variation in neonatal HIE outcomes is not explained only by insult severity or lesion size, but also by immune regulatory balance (23). It is also important to avoid oversimplifying the function of Treg. In this setting, their role may not be limited to dampening inflammation. By altering cytokine milieus, influencing innate immune cells, and potentially affecting tissue repair programs, Treg may shape the recovery trajectory itself (88). Thus, adaptive immune regulation in neonatal HIE should be viewed not only as a brake on injury, but as a determinant of how the injured brain reorganizes after the insult. This broader interpretation makes Treg particularly relevant to long-term outcome and supports including adaptive immunity as a distinct component of HIE pathophysiology.

### 4.3 Sexual dimorphism in neonatal HIE has an immune basis

Sex differences in neonatal hypoxic-ischemic brain injury have long been observed in both experimental and clinical settings, but the underlying mechanisms have remained incompletely understood (89). Emerging evidence now suggests that these differences are not solely attributable to intrinsic neuronal vulnerability or hormonal influences, but also have a significant immune basis. This is an important development because it reframes sex as an immunobiological variable rather than merely a background characteristic (24). Beckmann and colleagues demonstrated that Treg contribute to sexual dimorphism in neonatal HI brain injury, directly implicating adaptive immune regulation in sex-dependent outcome differences (23). This finding is complemented by the microglia-depletion study from Tsuji and colleagues, in which aggravation of injury after microglial depletion was predominantly observed in males (20). Considered together, these results support the idea that both innate and adaptive immune compartments participate in sex-specific injury responses in the developing brain.

Together, these observations suggest that sex-dependent differences in neonatal HIE may emerge from distinct immune-cell compositions, activation thresholds, or regulatory interactions rather than from a single isolated mechanism (24). In other words, the developing brain may exhibit sex-specific neuroimmune architectures that influence susceptibility to injury and capacity for recovery. This has direct implications for future therapy. If immune responses are sexually dimorphic, then immune-targeted interventions may not have identical efficacy across males and females, and sex-specific therapeutic strategies may ultimately become necessary (90). More broadly, these observations strengthen a central theme of this review: neonatal HIE is not simply an ischemic brain injury with inflammation layered on top. It is a developmentally specific neuroimmune disorder in which cell type, timing, and even biological sex shape how the immune response unfolds (26). Recognizing this complexity is essential for moving from descriptive inflammation to precision immunomodulation (91). These observations suggest that adaptive immunity should be considered an active component of neonatal HIE pathophysiology rather than a minor late-stage response. In particular, Treg may influence inflammatory balance, tissue repair, outcome heterogeneity, and sex-dependent vulnerability after neonatal hypoxic-ischemic injury. The proposed relationship between Treg-mediated immune regulation, innate–adaptive crosstalk, and sexual dimorphism is summarized in Figure 3.

**Figure 3:**
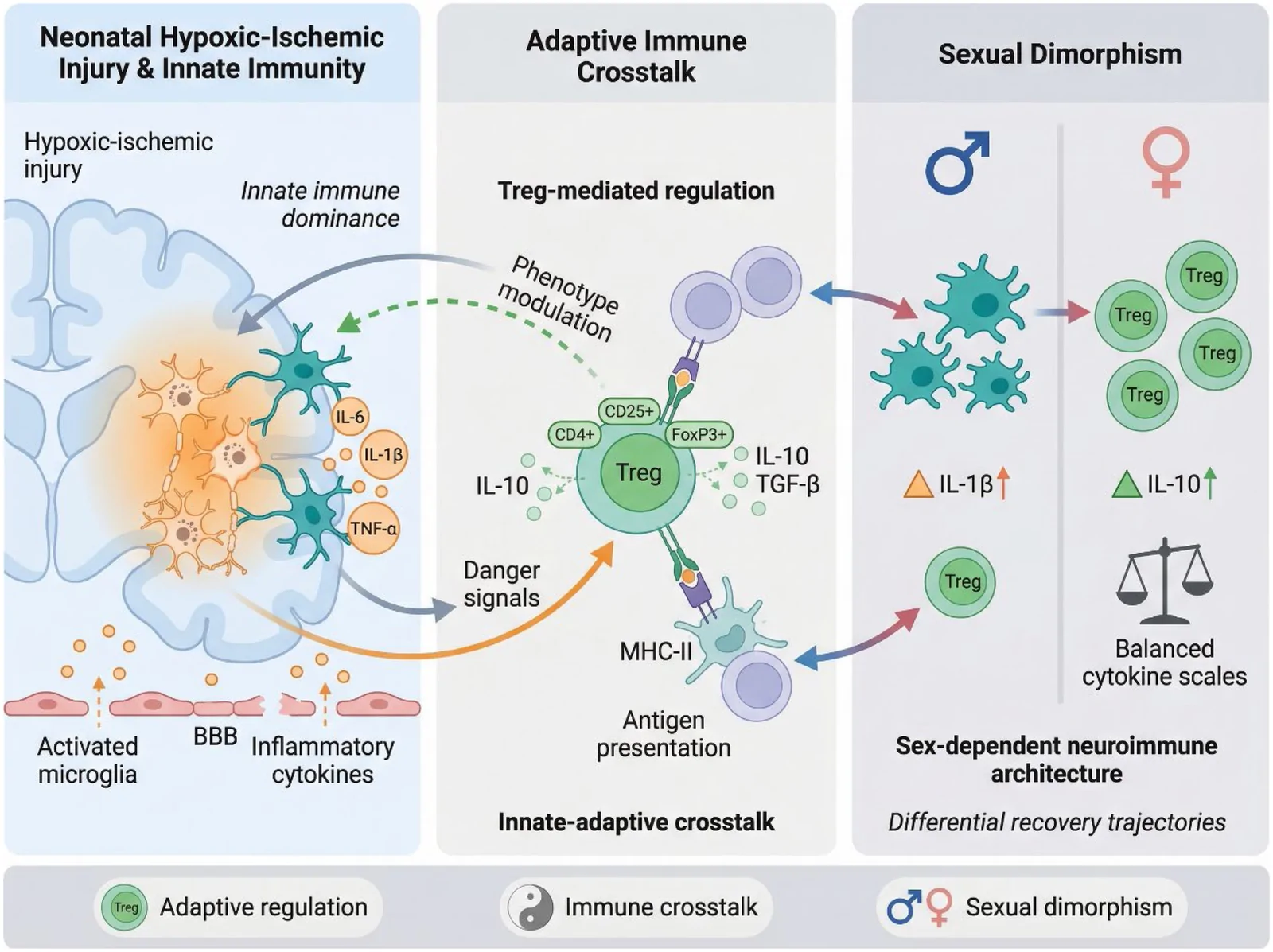
Adaptive immunity and sex-dependent neuroimmune responses in neonatal hypoxic-ischemic encephalopathy. Following hypoxic-ischemic injury, adaptive immune components, particularly regulatory T cells (Treg), contribute to neonatal HIE beyond innate immune activation alone. Treg interact with microglia and the post-ischemic inflammatory milieu to modulate immune balance, tissue repair, and outcome heterogeneity. These neuroimmune interactions also exhibit sex-dependent features, suggesting that sexual dimorphism in neonatal HIE has an important immune basis.

## 5 Cellular crosstalk in the injured neonatal brain

### 5.1 Immune cell-neuron crosstalk

In neonatal HIE, the interaction between immune cells and neurons is not limited to resident microglia. Although microglia are the earliest and most abundant immune sentinels in the injured neonatal brain, infiltrating monocytes, macrophage-like phagocytes, neutrophils, and adaptive immune cells may also enter or accumulate in the injured brain and interact with neuronal compartments directly or indirectly (92, 93). Therefore, neuron-directed immune crosstalk should be understood as a broader immune cell–neuron network rather than as a purely microglia–neuron axis.

Following hypoxic-ischemic injury, damaged neurons release danger-associated molecular patterns, ATP, oxidative stress-related signals, and intracellular contents that activate surrounding microglia and recruit or influence peripheral immune cells. Activated microglia can then alter neuronal survival through inflammatory cytokines, oxidative mediators, pyroptosis-associated signals, and altered phagocytic activity. Lv and colleagues showed that microglia-mediated pyroptosis contributes to neonatal hypoxic-ischemic brain damage by promoting inflammatory cytokine release, supporting a feed-forward loop between neuronal injury and innate immune activation. Similarly, microglial NF-κB activation has been linked to neuronal injury, whereas inhibition of microglial NF-κB attenuated hypoxic-ischemic damage in the immature brain (53, 94–97).

Beyond microglia, infiltrating and peripheral immune cells may further shape neuronal injury and repair. Monocytes can promote acute neuroinflammation and acquire pathological microglia-like features after neonatal hypoxic-ischemic brain injury (16), while long-term recruitment of peripheral immune cells has been observed in brain scars after neonatal insult. Phagocytic and myeloid populations may influence neurons through cytokine release, oxidative stress, antigen-processing functions, and IRF5-dependent inflammatory programming. Neutrophils may also contribute to neuronal damage through inflammatory mediators and neutrophil extracellular trap-related mechanisms, as inhibition of PAD4-mediated NET formation attenuated neonatal hypoxic-ischemic brain injury (98–101). These findings indicate that neuronal outcome is shaped not only by resident microglial activation but also by broader innate immune-cell entry and immune-effector functions.

Adaptive immune cells may also participate in neuron-directed immune regulation. Regulatory T cells have been implicated in sex-dependent differences after neonatal hypoxic-ischemic injury (102, 103). Although the precise mechanisms by which Treg influence neuronal survival remain incompletely defined, they may affect neuronal outcome indirectly by modulating cytokine balance, restraining excessive innate immune activation, and shaping the inflammatory milieu surrounding injured neurons. Thus, immune cell–neuron crosstalk in neonatal HIE should be viewed as a dynamic network involving resident microglia, infiltrating myeloid cells, neutrophils, and adaptive immune regulators. This expanded view has therapeutic implications. If neuronal damage is driven by a network of resident and infiltrating immune cells, then targeting microglia alone may be insufficient. Future strategies should consider how different immune-cell populations interact with neurons across acute, subacute, and remodeling phases, and should aim to suppress injurious immune–neuronal signaling while preserving debris clearance, immune resolution, and repair-supportive functions.

### 5.2 Microglia–myeloid crosstalk

The inflammatory environment in neonatal HIE is not shaped by microglia in isolation. Instead, resident microglia interact with a broader myeloid network that includes phagocytic and macrophage-like populations, together influencing the magnitude, persistence, and spatial distribution of post-ischemic inflammation (104–107). This broader perspective is important because it reframes neonatal HIE as a disorder of coordinated myeloid immune remodeling rather than a condition driven solely by resident brain immune cells. Microglia likely initiate part of this network response by rapidly sensing tissue injury and releasing inflammatory and chemotactic signals that influence other myeloid populations (26, 63). In turn, phagocytes and related myeloid cells can reinforce or reshape the inflammatory state of the injured brain through cytokine production, transcriptional programming, and interactions with local tissue compartments (101, 108). Al Mamun and colleagues showed that IRF5 signaling in phagocytes is detrimental in neonatal HIE, supporting the idea that myeloid inflammatory amplification is driven by defined transcriptional programs rather than by nonspecific immune activation alone (12). This finding suggests that microglia–myeloid crosstalk involves not only cell recruitment or coexistence, but also network-level coordination of pro-inflammatory vs. anti-inflammatory programming. This concept is further strengthened by the observation that therapeutic hypothermia alters microglia/macrophage polarization after neonatal hypoxic-ischemic injury. Seitz and colleagues showed that hypothermia modulates the temporal and spatial patterns of myeloid cell polarization, indicating that the benefits of standard treatment are not purely metabolic but also immunological (13). In other words, hypothermia appears to remodel the microglia–myeloid network itself rather than acting on a single cell type in isolation.

Taken together, these studies support the view that microglia–myeloid crosstalk is a central mechanism in the inflammatory evolution of neonatal HIE. Resident and non-resident myeloid populations likely cooperate to amplify injury in some contexts while contributing to resolution or repair in others. This makes the myeloid network an especially attractive target for therapeutic modulation, since reshaping its overall inflammatory balance may be more effective than focusing on a single immune compartment.

### 5.3 Microglia–adaptive immune crosstalk

Although neonatal HIE has classically been viewed as a disorder dominated by innate immune responses, adaptive immune components are increasingly recognized as functionally relevant rather than biologically negligible (109). This is particularly true for regulatory T cells, which may influence the local inflammatory milieu and thereby affect microglial phenotype, tissue damage, and recovery trajectory (110). Importantly, this means that adaptive immunity should not be considered a late or secondary addition to neonatal HIE pathophysiology, but part of the broader neuroimmune network that shapes outcome.

Beckmann and colleagues demonstrated that regulatory T cells contribute to sexual dimorphism in neonatal hypoxic-ischemic brain injury, providing direct evidence that adaptive immune regulation matters in this setting (23). Although the precise mechanisms of Treg–microglia interaction remain incompletely resolved, the conceptual framework is compelling: by modulating cytokine balance, inflammatory tone, and local cell-cell signaling, Treg may influence whether microglia adopt more injurious or more restrained functional states. In this sense, microglia–adaptive immune crosstalk may represent an additional regulatory layer superimposed on the myeloid inflammatory network. This perspective is especially important because it moves the field beyond the assumption that neonatal immune immaturity excludes meaningful adaptive immune participation. Instead, it suggests that the developing brain contains a uniquely configured immune environment in which even modest adaptive immune signals may have disproportionate effects on local inflammatory programming. Therefore, microglia–adaptive immune crosstalk should be considered a relevant component of neonatal HIE biology, particularly when discussing inter-individual variability, sex differences, and long-term recovery.

### 5.4 Immune crosstalk as a dynamic and phase-dependent network

One of the most important lessons from the current literature is that immune crosstalk in neonatal HIE is not static (25). Instead, it evolves across time, with different forms of cell-cell communication predominating at different phases of injury. In the acute phase, the dominant pattern is one of inflammatory escalation: injured neurons release danger signals, microglia become activated, pyroptosis-related pathways are engaged, and myeloid networks amplify tissue damage (22). During this period, crosstalk is largely organized around cell death, innate immune activation, and expansion of the lesion. In the subacute phase, however, the immune landscape becomes more complex. Microglial and myeloid phenotypes may begin to shift, inflammatory intensity may be partially restrained, and signals related to debris clearance, tissue containment, and early repair may emerge. This is also the phase in which therapeutic hypothermia appears to influence myeloid polarization, indicating that some immune interactions remain modifiable after the initial insult (13). In later phases, immune crosstalk may increasingly intersect with tissue remodeling, myelination, and neurodevelopmental outcome. At this stage, the consequences of earlier inflammatory states may persist as altered repair trajectories, white matter disruption, or developmental deficits (111). Thus, the significance of immune crosstalk in neonatal HIE extends beyond acute lesion formation and into the long-term shaping of brain maturation. Overall, the injured neonatal brain should be viewed as a dynamic neuroimmune ecosystem in which cell-cell interactions change over time and determine whether injury is amplified, contained, or maladaptively remodeled. Thus, immune crosstalk in neonatal HIE should be understood as a phase-dependent network rather than a fixed inflammatory reaction. During the acute, subacute, and remodeling phases, interactions among microglia, neurons, myeloid cells, and adaptive immune components may either amplify tissue injury or support containment and repair. This dynamic cellular crosstalk model is summarized in Figure 4.

**Figure 4:**
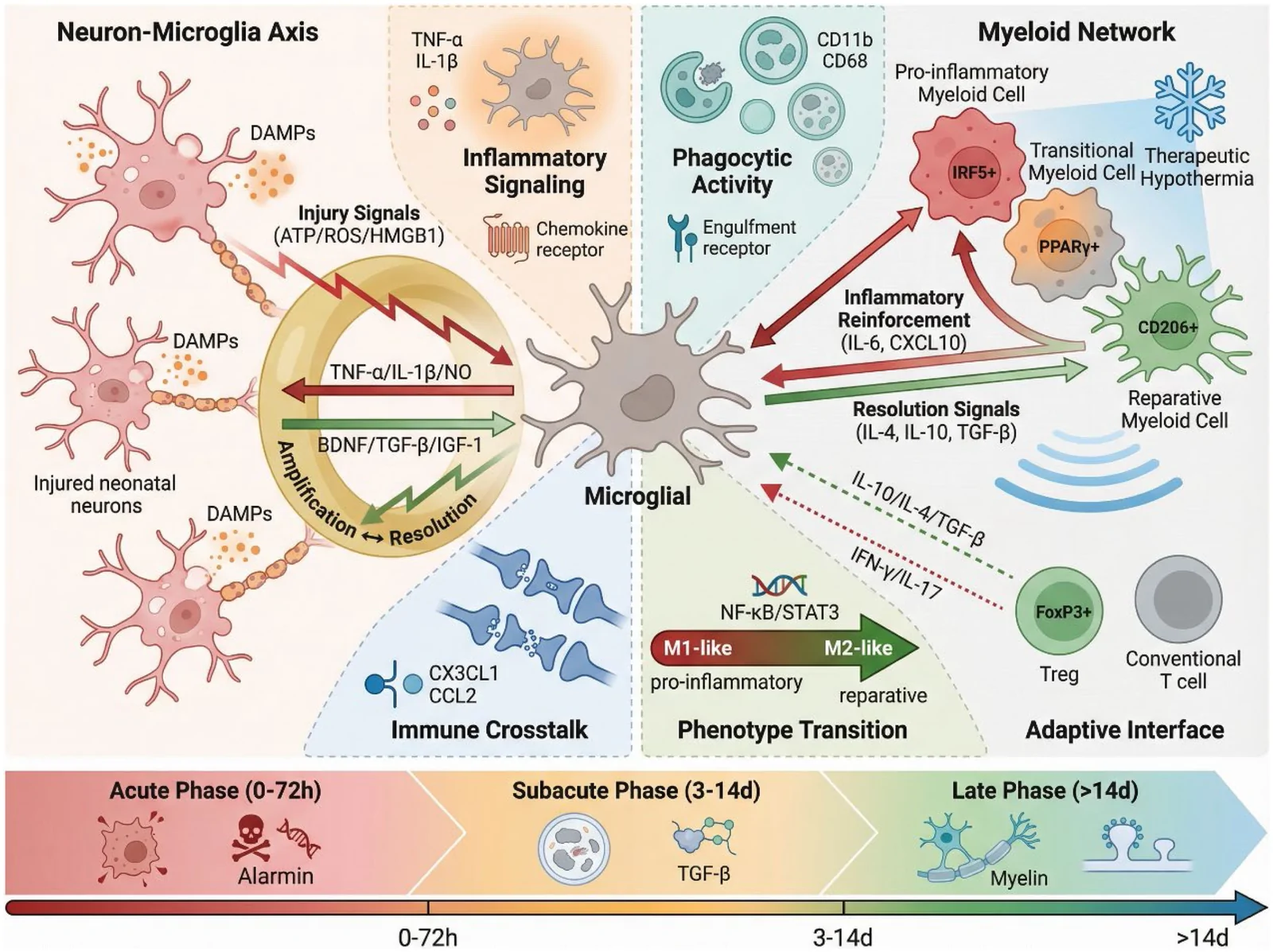
Immune cell–neuron crosstalk in the injured neonatal brain during hypoxic-ischemic encephalopathy. Hypoxic-ischemic injury activates resident microglia and promotes the recruitment or accumulation of peripheral immune cells, including monocytes/macrophage-like phagocytes, neutrophils, and adaptive immune regulators. These immune populations interact with injured neurons through danger signals, cytokines, oxidative mediators, phagocytosis, pyroptosis-related pathways, and inflammatory regulation. The network evolves across acute, subacute, and remodeling phases, thereby shaping injury amplification, tissue containment, repair, and long-term neurodevelopmental outcome.

## 6 Therapeutic targeting of immune cells in neonatal HIE

### 6.1 Why adjunctive immune targeting may complement hypothermia

Therapeutic hypothermia remains the standard of care for moderate to severe neonatal HIE, but its protective effect is incomplete. A substantial proportion of infants treated with hypothermia still experience death, disability, or long-term neurodevelopmental impairment. This limitation does not argue against hypothermia; rather, it highlights the need for adjunctive strategies that address mechanisms not fully controlled by cooling alone, including persistent inflammation, immune dysregulation, and impaired repair.

Importantly, the need for immune-targeted therapy does not arise in opposition to hypothermia, but because current evidence indicates that inflammation remains a major determinant of injury evolution despite standard treatment. Seitz and colleagues showed that hypothermia modulates myeloid cell polarization, implying that immune pathways are already part of the therapeutic landscape (13). However, this also means that the existing immune effects of hypothermia may be only partial, leaving room for additional interventions that more specifically reshape inflammatory signaling or repair programs. The translational rationale for adjunctive immune therapy is further supported by early clinical work. Cotten and colleagues conducted a pilot phase I trial of allogeneic umbilical cord tissue-derived mesenchymal stromal cells in neonates with HIE, highlighting the growing recognition that standard care may need to be supplemented by biologically targeted strategies (14). Together, these observations support a central premise of this section: immune targeting is needed not because hypothermia has failed completely, but because the pathobiology of neonatal HIE extends beyond what hypothermia alone can correct.

### 6.2 Targeting inflammatory signaling pathways in microglia and myeloid cells

A major therapeutic priority in neonatal HIE is the identification of inflammatory pathways that drive pathogenic activation in microglia and myeloid cells (112). The most promising near-term pharmacologic targets are not generic anti-inflammatory agents, but pathway-defined regulators of microglial and myeloid activation. This distinction matters because broad immunosuppression risks disrupting protective immune functions in the developing brain, whereas pathway-based targeting offers the possibility of selectively interrupting injurious programs.

NF-κB is one of the clearest examples. Zaghloul and colleagues demonstrated that inhibition of microglial NF-κB attenuated hypoxic-ischemic injury in the immature brain, supporting the view that this pathway acts as a functional driver of microglia-mediated damage rather than as a simple correlate of activation (54). Similarly, Tao and colleagues showed that Echinatin reduced inflammation and pyroptosis in hypoxic-ischemic brain damage through inhibition of TLR4/NF-κB signaling, linking upstream innate immune sensing to downstream inflammatory execution (48). Other pathways further broaden the target landscape. Aoki and colleagues identified LOX-1 as an upstream mediator of inflammatory activation in microglial cells under hypoxic-ischemic conditions, acting through p38-MAPK and NF-κB (53). Meanwhile, Al Mamun and colleagues showed that IRF5 functions as a detrimental transcriptional regulator in phagocytes, helping drive pro-inflammatory myeloid programming in neonatal HIE (12). These studies collectively indicate that the inflammatory architecture of neonatal HIE is organized through defined receptor- and transcription factor-based pathways that may be pharmacologically tractable.

Taken together, these data suggest that the most realistic near-term therapeutic strategy is not nonspecific inflammation suppression, but targeted interruption of selected signaling axes such as NF-κB, TLR4/MyD88/NF-κB, LOX-1/p38-MAPK/NF-κB, and IRF5-dependent myeloid programming. Such an approach is especially attractive in neonatal HIE because it offers a way to reduce damaging immune activation while preserving at least some of the protective and developmental functions of resident immune cells.

### 6.3 Cell-free immunomodulation: exosomes and extracellular vesicles

In parallel with pathway-based anti-inflammatory strategies, cell-free immunomodulatory approaches have emerged as an important therapeutic direction in neonatal HIE (31, 113). Among these, mesenchymal stromal cell-derived exosomes and extracellular vesicles are especially promising because they appear capable of reshaping the immune microenvironment without the practical and biological complexities associated with whole-cell transplantation (114, 115). This is a conceptually attractive model: rather than replacing damaged cells directly, EV-based therapies may work by reprogramming the inflammatory and reparative context of the injured brain.

Shu and colleagues showed that human bone marrow mesenchymal stem cell-derived exosomes protect against injury in neonatal hypoxic-ischemic brain damage by regulating the immune microenvironment (116). These data support the view that exosome therapy may act through immune microenvironment remodeling rather than through trophic support alone. Similarly, Labusek and colleagues reported that extracellular vesicles from immortalized mesenchymal stromal cells protect against neonatal hypoxic-ischemic brain injury, further supporting the idea that EVs can provide reproducible neuroprotection while also offering practical advantages in production and consistency (27). Compared with whole-cell therapy, EV-based interventions may offer better controllability, lower complexity of delivery, and a more defined extracellular mechanism of action. For this reason, they represent a leading example of “cell-free immunomodulation” in neonatal HIE. Their emergence is important not only because they expand therapeutic options, but because they shift the field toward microenvironment-centered treatment strategies that aim to rebalance immune signaling rather than simply block one pathway at a time.

### 6.4 Combination strategies with therapeutic hypothermia

Given that hypothermia remains the clinical foundation of neonatal HIE treatment, future immune-targeted therapies will likely need to function as adjunctive rather than replacement strategies. This is both biologically and clinically sensible. If hypothermia already modifies myeloid polarization and the inflammatory milieu, then additional therapies should ideally build on that altered landscape rather than ignore it. This principle is well illustrated by the work of Labusek and colleagues, who showed that hypothermia combined with extracellular vesicles from clonally expanded immortalized mesenchymal stromal cells improved neurodevelopmental impairment after neonatal hypoxic-ischemic brain injury (117). These results suggest that EV-based therapy may augment the benefits of standard care rather than compete with it. In other words, hypothermia may serve as a therapeutic platform onto which immune-modulating interventions can be added to enhance efficacy. More broadly, the concept of combination therapy reflects the biology of neonatal HIE itself. Because the disease includes metabolic failure, inflammatory amplification, cell death, and impaired repair, it is unlikely that any single intervention will fully address all relevant mechanisms. Adjunctive use of EVs, MSC-related products, or pathway-specific immunomodulators alongside hypothermia may therefore represent the most realistic route toward clinically meaningful improvement.

### 6.5 Clinical translation: from preclinical promise to early-phase trials

Perhaps the clearest sign that immune-targeted therapy in neonatal HIE is moving beyond proof-of-concept is the transition from experimental models to early clinical studies. Cotten and colleagues reported a pilot phase I trial of allogeneic umbilical cord tissue-derived mesenchymal stromal cells in neonates with HIE, marking an important milestone in translation (14). Although early-phase studies are necessarily focused on safety and feasibility, the existence of such a trial signals that immune-modulatory cell-based therapy is now being taken seriously as a clinical strategy rather than remaining confined to preclinical speculation.

At present, the major goals of clinical translation are to establish safety, determine feasibility in the context of standard hypothermia treatment, and identify preliminary signals of benefit. However, moving beyond early-phase feasibility will require more than simply replicating animal data in human infants. Future progress will depend on better patient stratification, more informative biomarkers of immune state, improved timing of intervention, and rational integration of adjunctive therapies with standard care. The broader implication is clear: MSC- and EV-based approaches have now advanced far enough that neonatal HIE immunotherapy can no longer be described as purely conceptual (118, 119). The field has entered a genuine translational stage. The challenge going forward is to convert promising neuroimmune mechanisms into precise, developmentally appropriate treatment strategies that improve outcome without disrupting essential immune and repair processes in the immature brain. Collectively, current therapeutic development in neonatal HIE is shifting from generalized neuroprotection toward immune-state-oriented intervention. Pathway-specific inhibition, EV/MSC-based immune microenvironment remodeling, and adjunctive combination strategies with hypothermia represent the major translational directions, although most remain at the preclinical or early clinical stage. These therapeutic strategies and their translational status are summarized in Table 3, and the overall treatment framework is shown in Figure 5.

**Table 3:** Emerging immune-targeted therapeutic strategies for neonatal HIE.

Therapeutic strategy	Main target/rationale	Main reported effect	Translational stage	Representative references
Therapeutic hypothermia (HT)	Standard care; modulates metabolism and immune polarization	Alters myeloid cell polarization and inflammatory distribution	Established clinical therapy	(13)
Microglial NF-κB inhibition	Pathway-defined suppression of injurious microglial activation	Reduces inflammatory signaling and attenuates brain injury	Preclinical	(54)
TLR4/NF-κB-targeted pharmacologic inhibition (e.g., Echinatin)	Blocks innate inflammatory signaling and pyroptosis	Reduces edema, infarct volume, inflammation, and pyroptosis markers	Preclinical	(48)
LOX-1/p38-MAPK/NF-κB targeting	Interrupts upstream inflammatory activation in microglia	Potentially restrains pro-inflammatory signaling cascades	Preclinical/mechanistic	(53)
IRF5-targeted myeloid reprogramming	Redirects detrimental phagocyte transcriptional programs	Reduces pro-inflammatory signaling and brain injury	Preclinical	(12)
MSC-derived exosomes/EVs	Cell-free immune microenvironment modulation	Neuroprotection through immune regulation and reparative signaling	Preclinical	(27, 116, 117)
HT + EV combination therapy	Adjunctive enhancement of standard care	Improves neurodevelopmental outcome beyond single strategies	Preclinical	(27, 117)
Umbilical cord tissue-derived MSCs	Clinical immunomodulatory cell therapy	Safety and feasibility explored in neonates with HIE	Early-phase clinical trial	(14)

**Figure 5:**
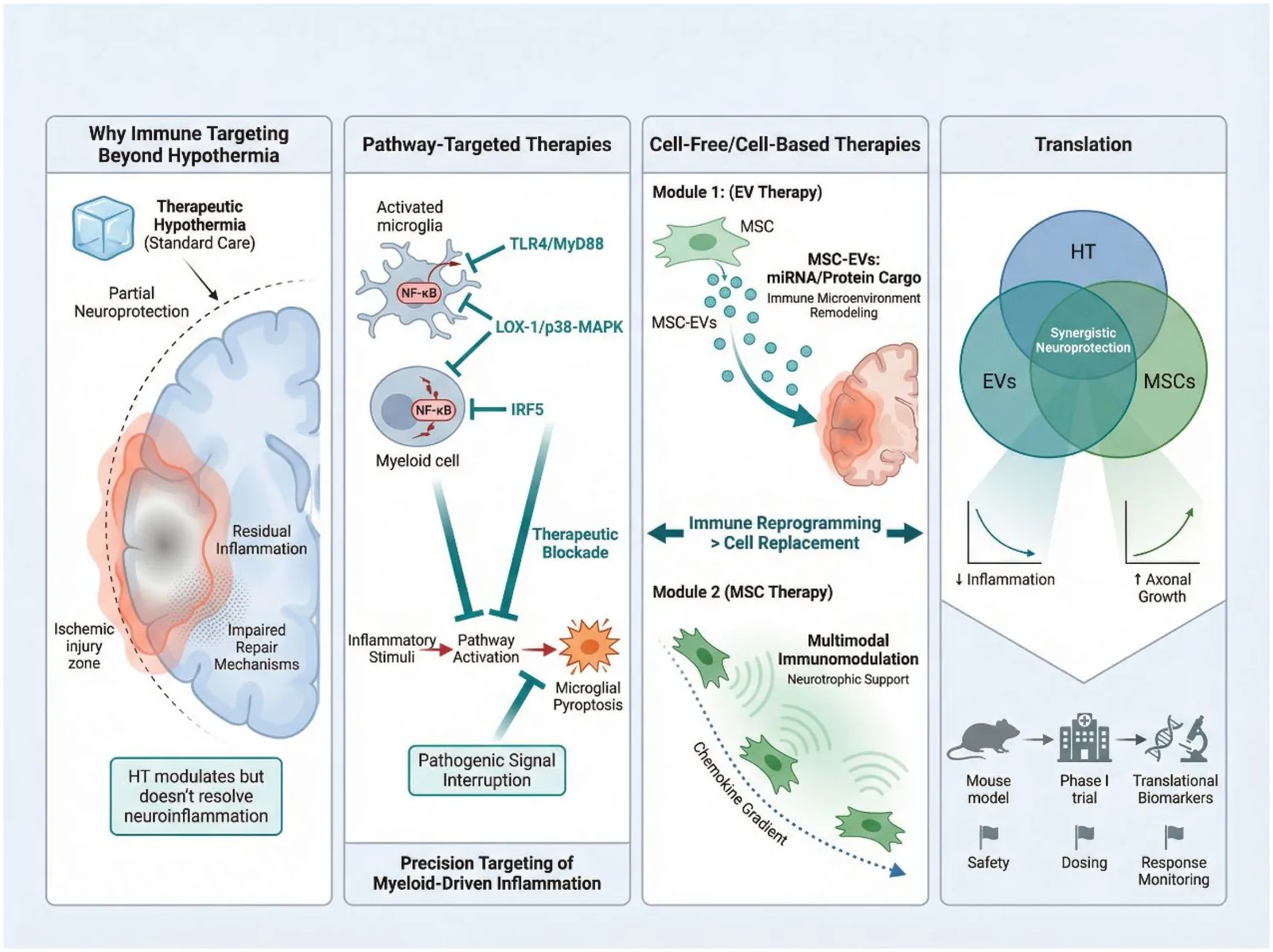
Immune-targeted therapeutic strategies in neonatal hypoxic-ischemic encephalopathy. Therapeutic hypothermia provides only partial protection in neonatal HIE, leaving persistent inflammation and impaired repair. Emerging strategies target pathogenic immune signaling in microglia and myeloid cells, while MSC- and EV-based approaches aim to remodel the immune microenvironment. These interventions are increasingly being developed as adjunctive therapies to hypothermia and are progressing from preclinical studies toward early clinical translation.

## 7 Challenges and future directions: from immune mechanisms to clinical translation

### 7.1 Defining phase- and phenotype-specific immune thresholds

A major challenge for future neonatal HIE research is to define when immune activation shifts from adaptive containment to maladaptive injury amplification. The preceding sections summarized evidence that microglia and myeloid cells can exert either protective or injurious effects depending on timing, phenotype, and local tissue context. Therefore, the unresolved question is no longer whether a given immune cell type is simply beneficial or detrimental, but how to identify the thresholds at which immune activity becomes excessive, prolonged, or misdirected. Future studies should move beyond broad cell-type labels and instead characterize immune responses according to activation magnitude, temporal phase, spatial localization, and functional state. This will require longitudinal profiling of immune-cell phenotypes across acute, subacute, and remodeling phases, ideally using approaches that integrate single-cell analysis, spatial mapping, cytokine dynamics, and neurodevelopmental outcomes. Such work may help distinguish immune programs that support debris clearance, injury containment, and repair from those that drive inflammasome activation, pyroptosis, cytokine amplification, and neuronal injury (32, 41, 42, 45, 120, 121). This distinction has direct therapeutic implications. Broad immune suppression may disrupt protective developmental and repair-related immune functions, whereas phase- and phenotype-specific modulation may allow injurious programs to be restrained while preserving immune activities required for recovery. Thus, a key future direction is to define actionable immune-state thresholds that can guide the timing and selection of adjunctive therapies.

### 7.2 Refining established neonatal models for spatially resolved neuroimmune analysis

Neonatal HIE research has a strong foundation in appropriately aged experimental models. Rather than being simply extrapolated from adult stroke, the field has long used neonatal rodent models as well as large-animal models, including fetal sheep and neonatal piglets, to define the temporal evolution of hypoxic-ischemic injury and to support clinical translation (122–125). These models have been central to the identification of latent and secondary phases of injury and have directly informed the development and clinical use of therapeutic hypothermia. Therefore, the key limitation is not the absence of developmentally relevant models, but the need to refine these established models for more detailed neuroimmune analysis. Future work should build on this foundation by integrating spatially resolved and longitudinal immune-state profiling into age-appropriate models. This is particularly important because the neonatal brain is characterized by ongoing synaptic refinement, myelination, evolving blood-brain barrier properties, and region-specific vulnerability. Immune responses may therefore differ across cortex, hippocampus, white matter, periventricular regions, lesion core, and lesion border. Large-animal models such as fetal sheep and neonatal piglets are especially valuable for bridging mechanistic and clinical questions, but they also need to be paired with modern approaches such as single-cell profiling, spatial transcriptomics, multiplex imaging, cytokine mapping, and clinically relevant outcome measures. Thus, the next stage of neonatal HIE modeling should not be framed as a replacement of existing models, but as a refinement of a well-established translational platform. The major goal is to connect injury phase, anatomical region, immune-cell state, and pathological phenotype in a way that can guide adjunctive therapies alongside standard hypothermia.

### 7.3 Biomarkers in neonatal HIE: clinical reality and translational limitations

Biomarker development in neonatal HIE should be discussed in relation to current clinical practice. Blood-based biomarkers have been investigated for decades, including markers of neuronal injury, glial activation, inflammation, oxidative stress, and systemic hypoxia (64, 126, 127). However, no blood-based biomarker is currently used routinely as a stand-alone tool for diagnosis, treatment selection, or outcome prediction in neonatal HIE. This limitation does not reflect a lack of candidate molecules, but rather fundamental challenges related to timing, biological specificity, assay standardization, feasibility, and validation across heterogeneous clinical cohorts. This issue is particularly important because therapeutic hypothermia must be initiated within a narrow early time window. In practice, eligibility for hypothermia is still determined primarily by clinical history, evidence of perinatal compromise, neurological examination, encephalopathy staging, blood gas or acid-base status, and electrophysiological assessment such as aEEG or EEG when available. These tools remain clinically actionable because they can be applied rapidly at the bedside, whereas many molecular biomarkers require sampling, processing, assay interpretation, and validation that may not fit the urgent decision-making window. The microRNA literature provides mechanistic insight into immune regulation and may eventually contribute to risk stratification or outcome prediction (128–131). For example, miR-210, miR-374a-5p, and miR-384 have been linked to microglial inflammation, inflammasome regulation, or biomarker potential. However, these candidates should not be presented as near-term clinical solutions. Their translational value will depend on whether they can be measured reproducibly, add information beyond standard clinical assessment and neurophysiology, and predict clinically meaningful outcomes within a time frame relevant to treatment decisions. Therefore, the future role of biomarkers in neonatal HIE is most likely complementary rather than substitutive. Biomarkers may help refine prognosis, support patient stratification, monitor immune-state evolution, or guide adjunctive therapies in research settings, but they should not be framed as replacing clinical assessment. Future studies should prioritize prospective validation, standardized sampling times, harmonized assays, integration with clinical and EEG/MRI data, and outcome-linked thresholds before biomarker-guided immunotherapy can be considered clinically actionable.

### 7.4 Critical appraisal of precision immunotherapy and combination strategies

The concept of precision immunotherapy in neonatal HIE should be interpreted cautiously. Although pathway-defined targeting is mechanistically attractive, neonatal HIE is not driven by a single linear inflammatory cascade. Instead, it involves overlapping and partially redundant mechanisms, including metabolic failure, excitotoxicity, oxidative stress, cytokine amplification, regulated cell death, glial activation, vascular dysfunction, and impaired developmental repair. Therefore, selective inhibition of a single regulator may be insufficient, particularly if parallel inflammatory or cell-death pathways compensate for the blocked mechanism. This limitation is important when considering targets such as IRF5. IRF5-dependent myeloid programming provides a useful example of how phagocyte transcriptional states can influence inflammatory amplification, but it should not be interpreted as a stand-alone therapeutic solution. Targeting IRF5 or similar regulators may be most useful as part of a broader strategy that considers injury phase, immune-cell state, compensatory signaling, and compatibility with standard hypothermia. By contrast, some of the therapies with greater translational promise, including MSC- and EV-based approaches, may act through broad immunomodulatory and trophic effects rather than through a narrowly defined molecular target (128, 132, 133). Their potential benefit may derive from coordinated effects on cytokine balance, glial activation, metabolic support, extracellular signaling, tissue repair, and immune resolution. In this sense, their relatively wide-ranging biological activity may be an advantage rather than a weakness in a disease characterized by multiple overlapping injury mechanisms. The clinical success of therapeutic hypothermia also supports this broader view. Hypothermia is unlikely to act through a single immune target; rather, its benefit probably reflects combined effects on metabolism, excitotoxicity, oxidative stress, cell death, inflammation, and repair. Therefore, the most biologically plausible future direction may not be narrow precision targeting alone, but precision-guided combination therapy. Such an approach would use biomarkers, timing, and immune-state assessment to determine when broad immunomodulation, MSC/EV-based therapy, or pathway-specific inhibition should be combined with standard hypothermia.

## 8 Conclusion

Neonatal hypoxic-ischemic encephalopathy should no longer be understood as a disorder defined solely by acute energy failure and neuronal loss. Instead, current evidence supports viewing HIE as a dynamic neuroimmune disorder in which immune cells actively shape injury progression, tissue response, and long-term developmental outcome. Microglia and broader myeloid populations are central drivers of this process, but their actions are not uniformly detrimental. Depending on timing, phenotype, and local context, these cells may either amplify inflammatory damage or contribute to containment and repair. At the same time, the neuroimmune landscape of neonatal HIE is more complex than previously appreciated. The growing recognition of regulatory T cells, adaptive immune involvement, and sex-dependent immune differences indicates that the immune response in neonatal HIE cannot be reduced to innate inflammation alone. Rather, it reflects a developmentally specific and highly interactive network in which cell type, timing, and biological sex all influence disease trajectory. Therapeutically, the field is moving beyond an exclusive reliance on hypothermia toward strategies aimed at reshaping the immune microenvironment. Pathway-specific targeting of microglial and myeloid activation, together with MSC- and EV-based immunomodulatory approaches, has opened a new translational landscape in which combination therapy is increasingly plausible. Early clinical studies suggest that this shift is no longer merely conceptual, but part of a genuine effort to develop adjunctive and precision-based interventions for neonatal HIE. Looking forward, the most important advances will likely come from three interconnected directions: a deeper understanding of timing, more refined analysis of cellular crosstalk, and the development of precision-targeted immunomodulation. Together, these efforts may help transform neonatal HIE management from generalized neuroprotection toward biologically informed neuroimmune therapy tailored to the injured developing brain.
